# Oocyte Family Trees: Old Branches or New Stems?

**DOI:** 10.1371/journal.pgen.1002848

**Published:** 2012-07-26

**Authors:** Dori C. Woods, Evelyn E. Telfer, Jonathan L. Tilly

**Affiliations:** 1 Vincent Center for Reproductive Biology, MGH Vincent Department of Obstetrics and Gynecology, Massachusetts General Hospital, Boston, Massachusetts, United States of America; 2 Department of Obstetrics, Gynecology and Reproductive Biology, Harvard Medical School, Boston, Massachusetts, United States of America; 3 Institute of Cell Biology and Centre for Integrative Physiology, University of Edinburgh, Edinburgh, United Kingdom; Stanford University School of Medicine, United States of America

The notion of a “biological clock” in women arises from the fact that oocytes progressively decline in number to the point of exhaustion as females get older, along with a decades-old dogmatic view that oocytes cannot be renewed in mammals after birth [1]. This latter thinking was challenged in 2004 when Tilly and colleagues [2], then others [3], reported that the rate of oocyte loss through follicular atresia and ovulation was much higher than the net rate of oocyte decline. This ignited an ongoing debate about whether the ovaries of adult mammals can form new oocytes and follicles [4]–[6]. Recent work demonstrating that oocyte-producing (oogonial) stem cells (OSCs; also referred to as female germline stem cells or fGSCs) exist in and can be isolated from ovaries of adult fish [7], [8], mice [2], [9]–[11], and even humans [11], [12] has led to new ideas about reproductive biological clocks. Earlier this year, a paper published in *PLoS Genetics* offered some of the most direct evidence to date that oogenesis in mice continues into adulthood under normal physiological conditions [13].

Shapiro and colleagues use a “molecular clock”—based on microsatellite mutations and a genetic trick to increase the mutation rate to ∼0.03 per cell per generation—to track the lineage relationships of individual cells, and reconstruct lineage trees in which inferred “depth”, or number of preceding mitotic cell divisions, is proportional to branch length [14]–[18]. Not surprisingly, the authors find that oocyte lineage trees are distinct from those of somatic cells; they then use the size and distribution of the lineage trees to estimate an initial oocyte progenitor pool of three to ten cells, similar to what has been estimated for the number of lineage-restricted primordial germ cell (PGC) precursor cells specified early in embryogenesis [19]. In addition, lineage trees from left and right ovaries are not distinct, which suggests there is substantial mixing of oocyte progenitors prior to the establishment of the two different ovary populations.

One of the most intriguing findings, though, is that oocytes exhibit a significant and progressive increase in depth as females age [13]. In other words, oocytes in older mice are derived from progenitor germ cells that have undergone more mitotic divisions than those that gave rise to oocytes in younger females. Two potential causal mechanisms are offered to explain this striking observation. The first, and the one that Reizel et al. dedicate the majority of their discussion to, is based on the “production-line hypothesis” first proposed by Henderson and Edwards in 1968 [20] as a potential explanation for the increase in oocyte chromosomal abnormalities and infertility observed with age. The production-line hypothesis states that oocytes in follicles are selected for maturation and ovulation throughout adult life in the same sequential order as their generation during fetal development. That is, oocytes matured and ovulated later in life theoretically committed to meiosis during embryonic development later than those germ cells that give rise to oocytes used earlier during adulthood.

Reizel et al. carry out simulations to depict how an embryonic meiotic production line could account for their observations, which they refer to as “depth-guided oocyte maturation”. However, a major problem with this idea is that the production-line hypothesis is based on differences in the timing of meiotic entry during embryogenesis, whereas depth of a given oocyte reflects the number of mitoses that occurred in the premeiotic germ cell (progenitor) that gave rise to that oocyte before it was formed. Proliferation of embryonic female germ cells in the mouse ceases at embryonic day 13.5 (e13.5) just prior to the onset of meiotic entry, which spans five days [21]–[23]. It is therefore unclear how oocytes formed at e18.5, and presumably matured later in life (viz. twelve months of age), would have significantly more depth than those formed only five days earlier (e13.5), and presumably matured first (viz. one month of age), in lieu of any additional rounds of germ cell mitosis between e13.5 and e18.5 (Figure 1).

**Figure 1:**
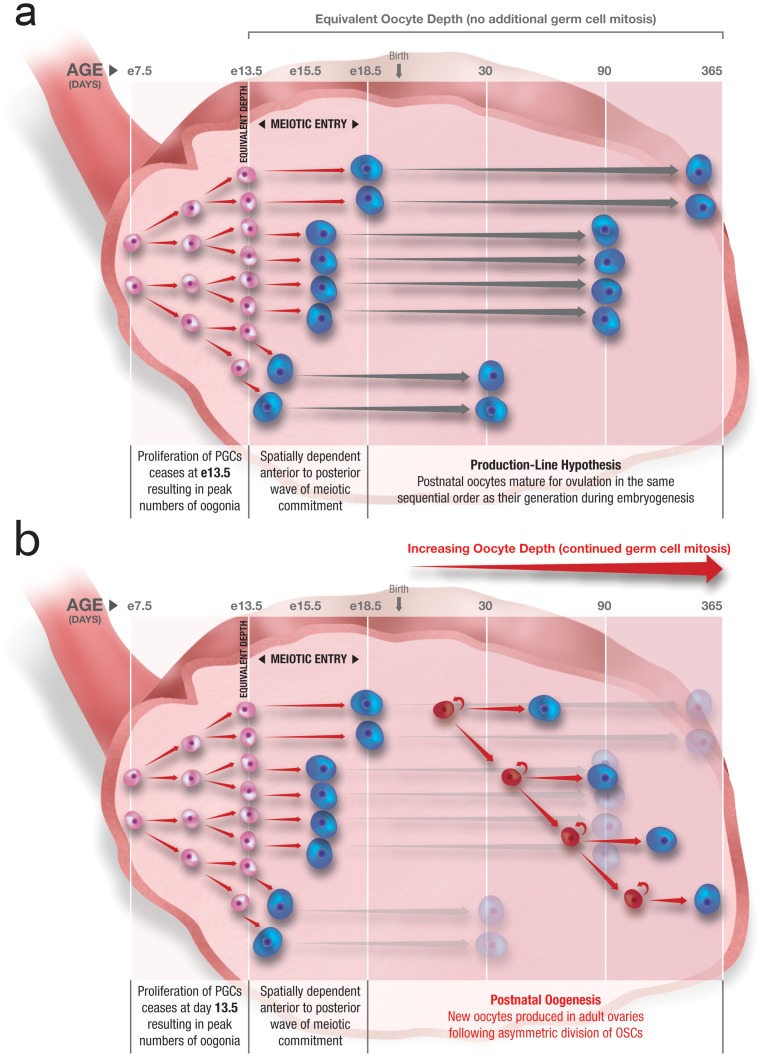
Postnatal oogenesis through ongoing oogonial stem cell (OSC) mitosis explains increasing oocyte depth with age. (a) Following primordial germ cell (PGC) expansion starting at embryonic day 7.5 (e7.5) in the mouse, proliferation of female germ cells (oogonia; *pink*) ceases at e13.5 concomitant with a 5-day period of germ cell meiotic commitment that drives formation of oocytes (*blue*); since all oocytes produced during this time are of equivalent “depth”, the production-line hypothesis of postnatal oocyte maturation cannot logically explain increasing oocyte depth as females age. (b) If continued proliferation of OSCs (*red*) and their subsequent differentiation into oocytes (*blue*) during postnatal life is superimposed on the production-line hypothesis, the emerging picture is consistent with a progressive increase in oocyte depth in females as they age.

We think a more logical explanation for the observations of Reizel et al. is that oocytes present in ovaries of older females arise from postnatal oogenesis, as successive mitotic divisions of OSCs with age give rise to new “deeper” oocytes. This suggestion, which Reizel et al. mention more in passing than as an explanation, is consistent with earlier work demonstrating the presence of rare proliferating germ cells in ovaries of mice during postnatal life [2]. These cells can be purified, continue to proliferate in vitro, and when transplanted into the ovaries of recipient mice generate fully functional eggs that fertilize to produce viable embryos and offspring [9], [11]. If oocytes in older female mice arise from actively dividing OSCs, those oocytes would have greater depth than oocytes from younger mice, since in younger females the oocyte pool would be derived either from embryonic PGCs or from postnatal OSCs that had undergone fewer mitotic divisions up to that point (Figure 1).

Interestingly, Reizel et al. also find that unilateral ovariectomy at one month of age results in an increase in oocyte depth in the remaining ovary when analyzed three months later compared with oocytes from age-matched control female mice possessing both ovaries [13]. Past studies with rodents have shown that following the removal of one ovary, compensatory ovulation occurs from the remaining ovary through increased follicle recruitment out of the immature follicle pool [24]–[29]. This leads to maintenance of a normal ovulatory quota in mice possessing only a single ovary, which persists for at least 75 weeks post-surgery [30]. Interestingly, despite the increased pull of follicles from the “single” ovarian reserve for long-term maintenance of normal ovulation rates, premature ovarian failure does not occur in unilaterally-ovariectomized mice [29]–[31], the follicle pool is not depleted at a greater rate [29]–[31], and there is no decline in the rate of follicle atresia which might provide a source of the additional immature follicles recruited for ovulatory growth [29]. Collectively, these historical data, coupled with the increase in depth of oocytes following unilateral ovariectomy reported by Reizel et al. [13], combine to make a compelling case for an increase in the rate of postnatal oogenesis in the remaining ovary as a very logical explanation for these findings.

In closing, the debate over whether mammals rely on OSCs and postnatal oocyte production for maintenance of ovarian function and fertility during adulthood is not yet settled. The recent purification of OSCs from ovaries of adult mice and women [9]–[11], and the fact that such cells, at least in mice, differentiate into fertilization-competent oocytes that produce viable embryos and offspring following intraovarian transplantation [9], [11], provide independent corroboration of their existence and functional potential. In addition, other work has reported the presence of dormant premeiotic germ cells in ovaries of aged female mice that resume the generation of new oocytes if moved into a young adult ovarian environment [32]. While these types of transplantation studies tell us what these newly discovered cells *can do*, it remains unclear what OSCs *are doing* in adult ovaries under normal physiological conditions. The recent work of Shapiro and colleagues is one of the first reports to offer experimental data consistent with a role for postnatal oocyte renewal in contributing to the reserve of ovarian follicles available for use in adult females as they age. Although unequivocal conclusions cannot be made at this point regarding the basis of the increase in oocyte depth described by Reizel et al. [13], their work is nonetheless an exciting and important addition to our understanding of reproductive biology and the origin of mammalian oocytes.

## References

[1] Zuckerman S (1951) The number of oocytes in the mature ovary. Rec Prog Horm Res 6: 63–108.

[2] Johnson J, Canning J, Kaneko T, Pru JK, Tilly JL (2004) Germline stem cells and follicular renewal in the postnatal mammalian ovary. Nature 428: 145–150. 15014492 10.1038/nature02316

[3] Kerr JB, Duckett R, Myers M, Britt KL, Mladenovska T, et al (2006) Quantification of healthy follicles in the neonatal and adult mouse ovary: evidence for maintenance of primordial follicle supply. Reproduction 132: 95–109. 16816336 10.1530/rep.1.01128

[4] Tilly JL, Niikura Y, Rueda BR (2009) The current status of evidence for and against postnatal oogenesis in mammals: a case of ovarian optimism versus pessimism? Biol Reprod 80: 2–12. 18753611 10.1095/biolreprod.108.069088 PMC2804806

[5] Tilly JL, Telfer EE (2009) Purification of germline stem cells from adult mammalian ovaries: a step closer towards control of the female biological clock? Mol Hum Reprod 15: 393–398. 19509111 10.1093/molehr/gap036 PMC2696346

[6] Woods DC, Tilly JL (2012) The next (re)generation of ovarian biology and fertility in women: is current science tomorrow's practice? Fertil Steril Epub ahead of print 6 June 2012. doi:10.1016/j.fertnstert.2012.05.005. 10.1016/j.fertnstert.2012.05.005 PMC4270270 22682028

[7] Nakamura S, Kobayashi K, Nishimura T, Higashijima S, Tanaka M (2010) Identification of germline stem cells in the ovary of the teleost medaka. Science 328: 1561–1563. 20488987 10.1126/science.1185473

[8] White YAR, Woods DC, Woods AW (2011) A transgenic zebrafish model of targeted oocyte ablation and de novo oogenesis. Dev Dynam 240: 1929–1937. 10.1002/dvdy.22695 21761478

[9] Zou K, Yuan Z, Yang Z, Luo H, Sun K, et al (2009) Production of offspring from a germline stem cell line derived from neonatal ovaries. Nat Cell Biol 11: 631–636. 19363485 10.1038/ncb1869

[10] Pacchiarotti J, Maki C, Ramos T, Marh J, Howerton K, et al (2010) Differentiation potential of germ line stem cells derived from the postnatal mouse ovary. Differentiation 79: 159–170. 20138422 10.1016/j.diff.2010.01.001

[11] White YAR, Woods DC, Takai Y, Ishihara O, Seki H, et al (2012) Oocyte formation by mitotically active germ cells purified from ovaries of reproductive-age women. Nat Med 18: 413–421. 22366948 10.1038/nm.2669 PMC3296965

[12] Telfer EE, Albertini DF (2012) The quest for human ovarian stem cells. Nat Med 18: 353–354. 22395699 10.1038/nm.2699

[13] Reizel Y, Itzkovitz S, Adar R, Elbaz J, Jinich A, et al (2012) Cell lineage analysis of the mammalian female germline. PLoS Genet 8: e1002477 doi:10.1371/journal.pgen.1002477. 22383887 10.1371/journal.pgen.1002477 PMC3285577

[14] Frumkin D, Wasserstrom A, Kaplan S, Feige U, Shapiro E (2005) Genomic variability within an organism exposes its cell lineage tree. PLoS Comput Biol 1: e50 doi:10.1371/journal.pcbi.0010050. 16261192 10.1371/journal.pcbi.0010050 PMC1274291

[15] Frumkin D, Wasserstrom A, Itzkovitz S, Stern T, Harmelin A, et al (2008) Cell lineage analysis of a mouse tumor. Cancer Res 68: 5924–5931. 18632647 10.1158/0008-5472.CAN-07-6216

[16] Wasserstrom A, Adar R, Shefer G, Frumkin D, Itzkovitz S, et al (2008) Reconstruction of cell lineage trees in mice. PLoS ONE 3: e1939 doi:10.1371/journal.pone.0001939. 18398465 10.1371/journal.pone.0001939 PMC2276688

[17] Wasserstrom A, Frumkin D, Adar R, Itzkovitz S, Stern T, et al (2008) Estimating cell depth from somatic mutations. PLoS Comput Biol 4: e1000058 doi:10.1371/journal.pcbi.1000058. 18404205 10.1371/journal.pcbi.1000058 PMC2275312

[18] Reizel YCI, Adar N, Itzkovitz R, Elbaz S, Maruvka J, et al (2011) Colon stem cell and crypt dynamics exposed by cell lineage analysis. PLoS Genet 7: e1002192 doi:10.1371/journal.pgen.1002192. 21829376 10.1371/journal.pgen.1002192 PMC3145618

[19] Ohinata Y, Payer B, O'Carroll D, Ancelin K, Ono Y, et al (2005) Blimp1 is a critical determinant of the germ cell lineage in mice. Nature 436: 207–213. 15937476 10.1038/nature03813

[20] Henderson SA, Edwards RG (1968) Chiasma frequency and maternal age in mammals. Nature 218: 22–28. 4230650 10.1038/218022a0

[21] Mintz B, Russell ES (1957) Gene-induced embryological modifications of primordial germ cells in the mouse. J Exp Zool 134: 207–238. 13428952 10.1002/jez.1401340202

[22] Tam PPL, Snow MHL (1981) Proliferation and migration of primordial germ cells during during compensatory growth in the mouse embryo. J Embryol Exp Morphol 64: 133–147. 7310300

[23] Menke DB, Koubova J, Page DC (2003) Sexual differentiation of germ cells in XX mouse gonads occurs in an anterior-to-posterior wave. Dev Biol 262: 303–312. 14550793 10.1016/s0012-1606(03)00391-9

[24] Hermreck AS, Greenwald GS (1964) The effects of unilateral ovariectomy on follicular maturation in the guinea pig. Anat Rec 148: 171–176. 14123501 10.1002/ar.1091480207

[25] McLaren A (1966) Regulation of ovulation rate after removal of one ovary in mice. Proc R Soc B Lond Biol Sci 166: 316–340. 10.1098/rspb.1966.0102 4382766

[26] Peppler RD, Greenwald GS (1970) Effects of unilateral ovariectomy on ovulation and cycle length in 4- and 5-day cycling rats. Am J Anat 127: 1–7. 5460936 10.1002/aja.1001270102

[27] Peppler RD, Greenwald GS (1970) Influence of unilateral ovariectomy on follicular development in cycling rats. Am J Anat 127: 9–14. 5460937 10.1002/aja.1001270103

[28] Chiras DD, Greenwald GS (1978) Acute effects of unilateral ovariectomy on follicular development in the cyclic hamster. J Reprod Fertil 52: 221–225. 564960 10.1530/jrf.0.0520221

[29] Baker TG, Challoner S, Burgoyne PS (1980) The number of oocytes and the rate of atresia in unilaterally ovariectomized mice up to 8 months after surgery. J Reprod Fertil 60: 449–456. 7431349 10.1530/jrf.0.0600449

[30] Biggers JD, Finn CA, McLaren A (1962) Long-term reproductive performance of female mice. I. Effect of removing one ovary. J Reprod Fertil 3: 303–312. 13869113 10.1530/jrf.0.0030303

[31] Jones EC, Krohn PL (1960) The effect of unilateral ovariectomy on the reproductive lifespan of mice. J Endocrinol 20: 129–134. 14407692 10.1677/joe.0.0200129

[32] Niikura Y, Niikura T, Tilly JL (2009) Aged mouse ovaries possess rare premeiotic germ cells that can generate oocytes following transplantation into a young host environment. Aging 1: 971–978. 20157580 10.18632/aging.100105 PMC2815754

